# Corticomedullary shunting after ischaemia and reperfusion in the porcine kidney?

**DOI:** 10.1186/s12882-022-02780-0

**Published:** 2022-04-15

**Authors:** Michael Rehling, Stine Gram Skjøth, Jørgen Frøkiær, Lene Elsebeth Nielsen, Christian Flø, Bente Jespersen, Anna Krarup Keller

**Affiliations:** 1 Department of Nuclear Medicine, PET-Center, Aarhus University Hospital, Aarhus, Denmark; 2 Department of Clinical Medicine, Aarhus University, Aarhus, Denmark; 3 Department of Renal Medicine, Aarhus University Hospital, Aarhus, Denmark; 4 Department of Urology, Aarhus University Hospital, Aarhus, Denmark

**Keywords:** Microspheres, Renal blood flow, Renal ischaemia, Renal perfusion, Renal redistribution, Renal shunts

## Abstract

**Background:**

Renal perfusion may redistribute from cortex to medulla during systemic hypovolaemia and after renal ischaemia for other reasons, but there is no consensus on this matter. We studied renal perfusion after renal ischaemia and reperfusion.

**Methods:**

Renal perfusion distribution was examined by use of ^153^Gadolinium-labeled microspheres (MS) after 2 h (hrs) and 4 h ischaemia of the pig kidney followed by 4 h of reperfusion. Intra-arterial injected MS are trapped in the glomeruli in renal cortex, which means that MS are not present in the medulla under normal physiological conditions.

**Results:**

Visual evaluation after reperfusion demonstrated that MS redistributed from the renal cortex to the medulla in 6 out of 16 pigs (38%) subjected to 4 h ischaemia and in one out of 18 pigs subjected to 2 h ischaemia. Central renal uptake of MS covering the medullary/total renal uptake was significantly higher in kidneys subjected to 4 h ischaemia compared with pigs subjected to 2 h ischaemia (69 ± 5% vs. 63 ± 1%, *p* < 0.001), and also significantly higher than in the contralateral kidney (69 ± 5% vs. 63 ± 2%, *p* < 0.001). Analysis of blood and urine demonstrated no presence of radioactivity.

**Conclusion:**

The study demonstrated the presence of MS in the renal medulla in response to renal ischaemia and reperfusion suggesting that severe ischaemia and reperfusion of the pig kidney leads to opening of functional shunts bypassing glomeruli.

## Background

Blood perfusion of the kidney is higher than the perfusion of any other organ when looking at flow in relation to the weight if the organ. In the human kidney, approximately 80% of the total perfusion distributes to the cortex providing only a minor fraction of the perfusion to the renal medulla [1], which also has a higher oxygen extraction during perfusion than the cortex. However, after severe systemic hypovolaemia, pale renal cortex with red highly perfused medulla is seen, and cortical necrosis with no regain of function is seen in some cases.

In 1947, Trueta and coworkers reported that renal blood flow appeared to be shunted to the renal medulla during haemorrhage or shock [2]. This phenomenon was termed “Cortical ischaemia with maintained blood flow through the medulla” [3, 4]. Redistribution of renal perfusion with relative cortical ischaemia is still an accepted outcome in response to haemorrhagic shock. Although almost 70 years have passed by since this original observation, there is still not a clear understanding on how this takes place [5–9].

Microspheres (MS) can be used to measure regional tissue blood flow [10]. Under normal physiological conditions intra-arterial injected microspheres are trapped in the glomeruli in the renal cortex. Thus, MS are not present in the renal medulla unless they have bypassed the glomeruli. We have developed a pig model where kidneys can be subjected to ischaemia by arterial clamping and subsequent reperfusion and we hypothesise that the number of MS present in the renal medulla is proportional to the number of functional shunts in the glomeruli. Thus, we examined the renal distribution of intra-arterial injected MS with the assumption that the presence of radio labeled MS in the renal medulla is a result of MS that have entered the renal medulla via shunts.

## Methods

The methods are described in detail elsewhere [11]. In brief, 34 female Danish landrace/Yorkshire pigs with a mean weight of 38 ± 2 kg were exposed to unilateral warm ischaemia by clamping the renal artery for 2 h (*n* = 18) or for 4 h (*n* = 16) followed by 4 h of reperfusion. Catheters were inserted in the left jugular vein, and the carotid artery for continuous monitoring of blood pressure, infusion of drugs and fluid. Baby feeding tubes were placed in the ureters to collect urine. The aorta was catheterised through the femoral artery and the catheter tip for MS injection was placed in the aortic arch. After 4 h of reperfusion, renal perfusion was estimated by injection of 15–20 MBq of ^153^Gadolinium-labeled MS with a diameter of 15 µm. After termination and nephrectomy, the renal distribution of MS was studied in absolute counts over each kidney using a gamma-camera (BrightView, Philips Medical Systems, San Jose, CA, USA, 2009). Total renal counts and counts over the central renal areas including medulla were estimated by computer drawing of the region of the entire kidney and over the central 75% of the kidney. All counts were corrected for background radiation and acquisition time, and normalised to the amount of injected tracer.

Based on the visual perfusion redistribution of the scintigram, each kidney was graded from 0 – 3 in a blinded way by two observers: Grade 0: Homogeneous pattern similar to control kidneys. Grade 1: Weaker activity, but still homogeneous. Grade 2: Showing a pattern, identifying the medulla. Grade 3: Showing a pattern, clearly showing the medullary architecture and the anatomical outline of calyces.

### Statistics

For statistical analysis an open-source statistical package from SciPy was used [12]. (version 0.17.1, www.scipy.org, 2016). Observer agreement were assessed using Cohen's Kappa statistic. Continuous data are expressed as mean ± standard deviation, geometric mean with 95% confidence interval (CI) or number with percentages, and with 95% CI when appropriate. Differences between groups were tested using Welsh T-test. Mann–Whitney U tests were used to compare independent groups when data were not normally distributed. A difference was considered significant when *P* < 0.05.

## Results

The weight of the kidneys subjected to 4 h of ischaemia was significantly higher than the weight of the kidneys subjected to 2 h of ischaemia (*p* < 0.005) (Table 1). In both groups the renal weight was significantly higher than the weight of the contralateral non-ischaemic kidney (*p* < 0.001). At baseline (60 min), we found a mean single kidney GFR value of 30.2 ± 8.8 ml/min with no significant difference between groups [11]. During reperfusion the regain in GFR was close to zero.

**Table 1:** Kidney weight and michrosphere uptake

	Ischaemia 2 hrs (*n*=18)		Ischaemia 4 hrs (*n*=16)		*p*-values
	Ischaemic kidney	Contralateral kidney	Ischaemic kidney	Contralateral kidney	Ischaemic vs Contralateral kidney
Kidney weight*(gram)*	109 ± 13*	90 ± 13	136 ± 28*	101 ± 17	0.0023**
Absolute uptake of microspheres*(cps MBq-1)*	3.8 ± 1.5	4.1 ± 1.8	1.6 ± 1.5*	4.0 ± 1.3	0.0002**
Relative uptake of microspheres*(percent of total uptake)*	48 ± 14	52 ± 14	29 ± 19*	71 ± 19	0.0028**
Renal uptake in central 75 % including renal medulla*(percent of whole kidney uptake)*	63*	62	69*	63	0.0002**

The table gives the weight of the two kidneys and the absolute and relative uptake of microspheres in the kidney after two and four hours of unilateral renal ischaemia followed by reperfusion for four hours

All numbers as means ± SD. * denotes significant difference between ischaemic and contralateral kidneys. ** denotes significant difference between the ischaemic kidney at 2 and 4 hours ischaemia

The total renal uptake of MS as an indicator of blood flow was significantly lower after 4 h ischaemia than after 2 h ischaemia (*p* < 0.001). After 4 h ischaemia the MS uptake was also significantly lower than the uptake of the contralateral kidney (*p* < 0.001). The renal MS uptake after 2 h ischaemia did not differ significantly from the uptake of the contralateral kidney (*p* = 0.5) (Table 1).

Uptake of MS in the 75% central renal area including the medulla measured in per cent of uptake in the total kidney was significantly higher in kidneys subjected to 4 h compared to 2 h ischaemia (*p* < 0.001). In kidneys subjected to renal ischaemia for 4 h it was also significantly higher than in the contralateral kidney (*p* < 0.001) (Table 1).

**Fig. 1:**
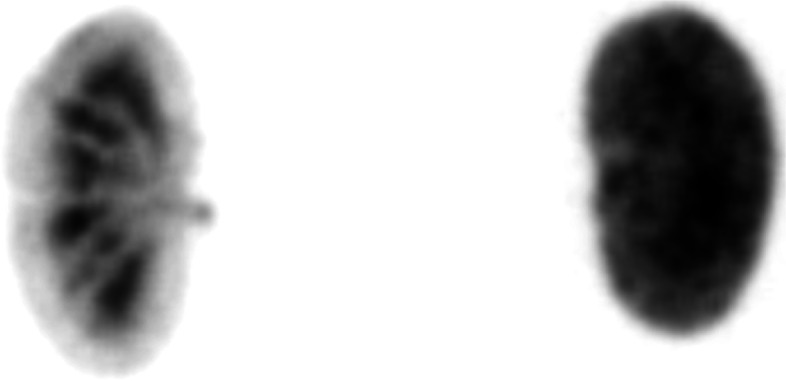
Distribution of radio-labeled microspheres in a pig after renal ischaemia and reperfusion In comparison to the non-ischaemic right kidney, the left kidney shows an increased uptake of radio-labeled microspheres in renal medulla relative to the total kidney

**Fig. 2:**
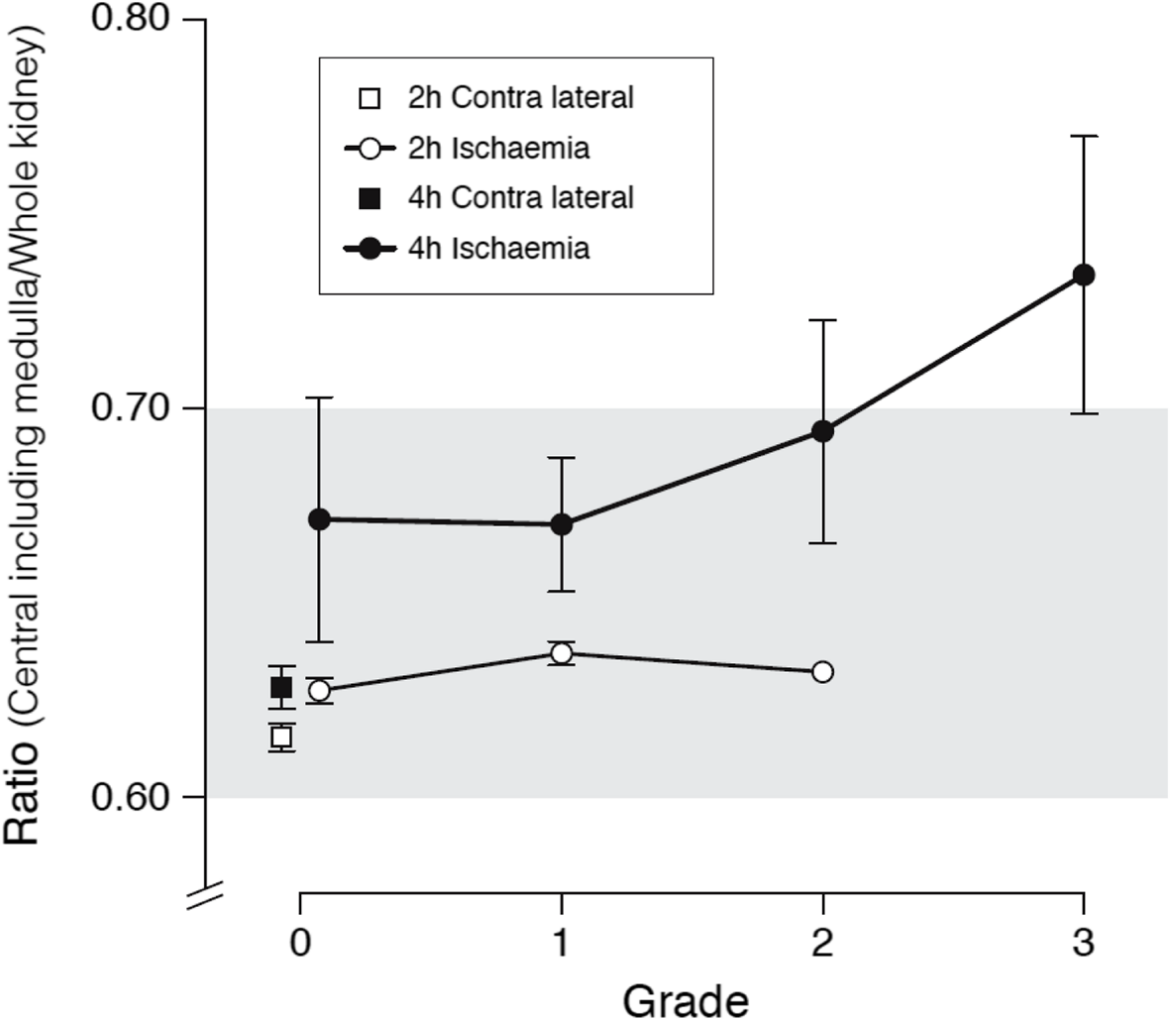
Comparison of methods for the study of distribution of radio-labeled microspheresThe figure shows a positive correlation between visual distribution score grade 1–3 and the measured fraction of microspheres in the central kidney including medulla relative to the entire kidney (*r*2 = 0.98). Differences between 2 and 4 h ischaemia is marginally significant (*p* ~ 0.08). Grade 0 is a homogeneous pattern similar to control kidneys and Grade 3 is a pattern clearly showing the medullary architecture and the anatomical outline of calyces

Redistribution of MS from cortex to medulla as assessed by blinded visual evaluation of the scintigrams showed redistribution in 6 out of 16 pigs (38%) subjected to ischaemia for 4 h, and in one out of 18 pigs (5%) with ischaemia for 2 h. Figure 1 shows an example of renal MS redistribution after 4 h ischaemia. The scintigram from the ischaemic side shows high activity in the medulla and faint activity in the renal cortex. Figure 2 shows that the higher the perfusion redistribution grade of the kidney, the higher uptake of MS in the central renal area including the medulla relative to uptake in total kidney.

Analysis of blood and urine samples demonstrated that no detectable radioactivity was present in these samples.

## Discussion

The present study suggests that radioactive labeled microspheres (MS) can identify corticomedullary shunts after ischaemia and reperfusion of the human like polypapillary porcine kidney.

We studied renal blood flow by use of uptake of MS after unilateral renal ischaemia for 2 and 4 h followed by 4 h of observation after reperfusion. After intra-arterial injection of MS the absolute renal uptake of MS was significantly reduced in the kidneys subjected to 4 h total ischaemia. The uptake of MS after 2 h of renal ischaemia did not differ significantly from the uptake in the contralateral non-ischaemic kidney. After 4 h ischaemia, there was a relatively high uptake of MS in the central area of the kidney including the renal medulla both on the scintigrams and calculated from the absolute values. These observations may be explained by the special anatomy of blood supply to the kidneys. Blood supply to the nephron is maintained by two vascular systems organised in a serial manner: the capillaries in the glomeruli and the tubular capillaries in the renal medulla [1]. Thus, intra-arterial injected MS reach the renal cortex with the arterial blood, and in the glomeruli they are trapped due to their diameter of 15 µm and thereby separated from the blood stream. Thus, measuring total renal blood flow may be done from the number of MS in the whole kidney, whereas regional distribution of MS is unreliable in calculation of the relative distribution of renal perfusion. However, this normal trapping of MS in the glomeruli explained by renal anatomy makes it possible to quantify functional active shunts from the number of MS present in the renal medulla. In contrast to the early anatomical studie by Trueta and others [2] we made a physiological study with MS as a tracer. We measured uptake of radiolabelled MS on planar images by use of a 2-dimensional technique. We did not have access to SPECT/CT images in these pig studies. Therefore, due to anatomical overlap of medulla and cortex the uptake in renal medulla will be overestimated, but the uptake in the central 75% includes the total medulla. Our results are consistent with the opening of corticomedullary shunts in the ischaemic kidney. However, we cannot exclude that vasodilation in cortical juxtamedullary afferent arterioles after long-term ischaemia could explain our findings, although we believe that our 15 µm MS would not be able to pass the capillaries in glomeruli. Our visualisation technique seems to indicate presence of MS in medulla and not in the juxtamedullary cortex.

Our findings of corticomedullary shunts in severely ischaemic kidneys are in agreement with a resent published study by Schutter and coworkers [13]. By use of MRI they assessed the renal flow over time during normothermic machine perfusion (NMP) in porcine kidneys and human kidneys discarded for transplantation. Interestingly, they demonstrated by use or MRI, that the regional renal flow is entirely different from the total renal flow, and they found for all kidneys that the central region and medulla of the kidney was predominantely perfused initially, while the cortex reached a dominant perfusion state after 1–2 h, normalizing the flow distribution in the kidney. In addition, when they added a period of NMP hypoperfusion, demonstration a intrarenal shift of perfusion from the cortical area to the medullar regions. In their study, normal flow distribution was achieved in all kidneys, which was not the case in our study where the corticomedullary shunt remained in 38% and 5% of the kidneys subjected to 4 and 2 h ischemia followed by reperfusion. These are most likely the most damaged kidneys but it would have been interesting to see if these kidney could have improved had they been subjected to NMP instead of in situ reperfusion. From other experimental studies we know, that even severely damaged porcine kidneys with 75 min warm ischemia can be successfully transplanted in a survival model [14]. Prolonged warm ischemia remains a challenge in donation after circulatory death and a better understanding of the cotticomedullary shunts may be of benefit.

## Conclusion

In conclusion, the study showed the presence of radiolabeled MS in the renal medulla in response to severe renal ischaemia suggesting that this leads after reperfusion to opening of functional shunts bypassing the glomeruli. Radiolabeled MS have been used for decades for the measurement of organ blood flow and perfusion and the present results point to functional intrarenal shunts, which have been suggested for decades, but not verified so far.

## Data Availability

The datasets generated and analysed during the current study are included in this published article, and are available from the corresponding author on reasonable request.

## References

[1] Munger K, Kost C, Brenner B, Maddox D, Taal M, Chertow G, Marsden P, Skorecki K, Yu Y, Brenner B (2012). The renal circulations and glomerular ultrafiltration. Brenner and Rectors, The Kidney.

[2] Trueta J, Barclay A, Daniel P (1947). Studies of the renal circulation.

[3] Daniel P, Peabody C, Prichard M (1952). Cortical ischaemia of the kidney with maintained blood flow through the medulla. Q J Exp Physiol Cogn Med Sci.

[4] Daniel P, Peabody C, Prichard M (1951). Observations on the circulation through the cortex and the medulla of the kidney. Q J Exp Physiol Cogn Med Sc.

[5] Lilienfields L, Maganzini H, Bauer M (1961). Blood flow in the renal medulla. Circ Res.

[6] Spinelli FR, Wirz H, Brucher C, Pehling G (1972). Non-existence of shunts between afferent and efferent arterioles of juxtamedullary glomeruli in dog and rat kidneys. Nephron.

[7] Stone AM, Stein T, LaFortune J, Wise L (1979). Changes in intrarenal blood flow during sepsis. Surg Gynecol Obstet.

[8] Greenfield SP, Lewis W, Perry B, Wan J, Morin F (1995). Regional renal blood flow measurements using radioactive microspheres in a chronic porcine model with unilateral vesicoureteral reflux. J Urol.

[9] Langenberg C, Bellomo R, May C, Wan L, Egi M, Morgera S (2005). Renal blood flow in sepsis. Crit Care.

[10] Peters A, Myers M, Peters A, Myers M (2003). Measurement of blood flow. Physiological measurements with Radionuclides in Clinical Practice.

[11] Pedersen SS, Keller AK, Nielsen MK, Jespersen B, Falborg L, Rasmussen JT (2013). Cell injury after ischemia and reperfusion in the porcine kidney evaluated by radiolabelled microspheres, sestamibi, and lactadherin. EJNMMI Res.

[12] Jones E, Oliphant T, Peterson P. SciPy. Open source scientific tools for Python. 2001. Available from: http://www.scipy.org/. Accessed Sept 2013.

[13] Schutter R, Lantiga VA, Hamelink TL, Pool MBF, Varsseveld OCV, Potze JH (2021). Magnetic resonance imaging assessment of renal flow distribution patterns during ex vivo normothermic machine perfusion in porcine and human kidneys. Transpl Int.

[14] Lohmann S, Eijken M, Møldrup U, Møller BK, Hunter J, Moers C, et. al.: Ex Vivo Administration of Mesenchymal Stromal Cells in Kidney Grafts Against Ischemia-reperfusion Injury-Effective Delivery Without Kidney Function Improvement Posttransplant.Transplantation. 2021 1;105(3):517–528. doi: 10.1097/TP.0000000000003429. 10.1097/TP.0000000000003429 32956281

